# Supplementary material for: Polyketide synthase gene clusters in the ericoid mycorrhizal fungus Oidiodendron maius: genome-wide prediction, expression profiling, and functional characterization of knockout mutants
Source: Front Plant Sci. 2026 Sep 16;17:1924430. doi: 10.3389/fpls.2026.1924430 (PMC13623903; doi:10.3389/fpls.2026.1924430)
Supplement: Supplementary file 1 [file SupplementaryFile1.zip › Supplementary Materials/Supplementary Folder 1.zip!/Oidma1_AssemblyScaffolds_Repeatmasked/knownclusterblast/cluster51/jgi.p_Oidma1_61609_mibig_hits.html]

| MIBiG Protein | Description | MIBiG Cluster | MiBiG Product | % ID | % Coverage | BLAST Score | E-value |
| --- | --- | --- | --- | --- | --- | --- | --- |
| ADI24929.1 | VrtD | BGC0000168 | Viridicatumtoxin\_biosynthetic\_gene\_cluster | 50 | 100.586510264 | 329 | 4.7e-90 |
| BAD86799.1 | geranylgeranyl\_diphosphate\_synthase | BGC0000286 | Viguiepinol\_biosynthetic\_gene\_cluster | 26 | 77.7126099707 | 82 | 7.6e-16 |
